# Localized Rejuvenation of a Crystal Mush Recorded in Zircon Temporal and Compositional Variation at the Lassen Volcanic Center, Northern California

**DOI:** 10.1371/journal.pone.0113157

**Published:** 2014-12-03

**Authors:** Erik W. Klemetti, Michael A. Clynne

**Affiliations:** 1 Department of Geosciences, Denison University, Granville OH 43023, United States of America; 2 U.S. Geological Survey, Volcano Science Center, Menlo Park CA 94025, United States of America; University of Oxford, United Kingdom

## Abstract

Zircon ages and trace element compositions from recent silicic eruptions in the Lassen Volcanic Center (LVC) allow for an evaluation of the timing and conditions of rejuvenation (reheating and mobilization of crystals) within the LVC magmatic system. The LVC is the southernmost active Cascade volcano and, prior to the 1980 eruption of Mount St. Helens, was the site of the only eruption in the Cascade arc during the last century. The three most recent silicic eruptions from the LVC were very small to moderate-sized lava flows and domes of dacite (1915 and 27 ka eruptions of Lassen Peak) and rhyodacite (1.1 ka eruption of Chaos Crags). These eruptions produced mixed and mingled lavas that contain a diverse crystal cargo, including zircon. ^238^U-^230^Th model ages from interior and surface analyses of zircon reveal ages from ∼17 ka to secular equilibrium (>350 ka), with most zircon crystallizing during a period between ∼60–200 ka. These data support a model for localized rejuvenation of crystal mush beneath the LVC. This crystal mush evidently is the remnant of magmatism that ended ∼190 ka. Most zircon are thought to have been captured from “cold storage” in the crystal mush (670–725°C, Hf >10,000 ppm, Eu/Eu* 0.25–0.4) locally remobilized by intrusion of mafic magma. A smaller population of zircon (>730°C, Hf <10,000 ppm, Eu/Eu* >0.4) grew in, and are captured from, rejuvenation zones. These data suggest the dominant method to produce eruptible melt within the LVC is small-scale, local rejuvenation of the crystal mush accompanied by magma mixing and mingling. Based on zircon stability, the time required to heat, erupt and then cool to background conditions is relatively short, lasting a maximum of 10 s–1000 s years. Rejuvenation events in the LVC are ephemeral and permit eruption within an otherwise waning and cooling magmatic body.

## Introduction

The connection between the volcanic and plutonic record has always been elusive. While volcanic rocks from polygenetic magmatic systems are the result of periodic tapping of an active magmatic body, plutonic rocks are an integrated history potentially spanning the life of a magmatic system. Attempting to reconcile the processes that are seen in plutonic structures versus mineral ages and compositions of volcanic rocks can problematic [1], [2], [3], [4], [5], as many processes that occur during the consolidation of a pluton may have no manifestation in erupted material. However, examining the temporal and compositional populations of refractory minerals like zircon, can bridge the integrated plutonic and the punctuated volcanic records.

This is especially important in understanding long-lived magmatic systems, where prolonged injections of magma into the crust are building new plutons. These systems with histories of magmatism over 10^5^–10^6^ years are places where repeated rejuvenation of crystal mushes (or incipient plutons) drives the evolution of magma [6], [7], . In many cases, our understanding of rejuvenation is based on large-volume centers, such as the Yellowstone caldera [9], [10], Toba [11], the Fish Canyon Tuff [12], the Okataina Caldera Complex [4], [13], [14], [15], the Bishop Tuff [16], [17] or the Southern Rocky Mountain Volcanic Field [18]. In these systems, rejuvenation affects a large volume of the intrusive body. However, in studies of plutons and batholiths, the evidence of rejuvenation (e.g., [19], [20], [21], [22]) is more localized, with only small parts of the crystallizing body taking part in thermal rejuvenation and new magma movement. In both cases, the timescales of rejuvenation are not well constrained.

In studies of smaller volcanic systems, the case of rejuvenation suggests that short periods of time are required for remobilizing crystals contained in a subvolcanic crystal mush. Andesitic systems such as Soufriere Hills show remobilization of diverse major phase populations during new influxes of magma that may only take 10^1^–10^3^ years [23], [24], [25]. More silicic systems such as Mount St. Helens or the Devil's Hills and Rock Mesa on Oregon's South Sister show a mix of younger major phases and older trace phases [8], [26], [27].

Zircon extracted from the 1915 dacite of Lassen Peak, ∼1.1 ka rhyodacite of Chaos Crags and ∼27 ka dacite of Lassen Peak in the Lassen Volcanic Center (LVC) in the Cascades of northern California capture the rejuvenation of an evolving magmatic body. Most of these zircon formed from ∼200 to 60 ka, encompassing the end of the Bumpass sequence (350-193 ka), a period of volcanic quiescence (193-90 ka) and the start of the Twin Lakes and Eagle Peak sequences (<90 ka, all sequence dates from [28]), ceasing at ∼17–20 ka. The temporal and compositional distribution of zircon from these three most recent silicic eruptions within the LVC record two magmatic states: (1) zircon forming in a plagioclase-fractionation-dominated cooling intrusion derived from Bumpass sequence magmatism and (2) zircon forming in localized zones of rejuvenation within the intrusion during end of the eruptive hiatus and across the Twin Lakes and Eagle Peak sequences. These data capture the small-scale, localized rejuvenation of a crystallizing magma body on timescales of hundreds to thousands of years. These rejuvenation events allow for recycling and movement of phenocrysts (crystallized from active magma), autocrysts (crystallized from active magma in trace proportions), and antecrysts (scavenged crystals from previous magmatic events) along pathways for rejuvenated melts. Understanding these rejuvenation events will help bring together the volcanic and plutonic records of arc magmatism.

## Setting

**Figure 1:**
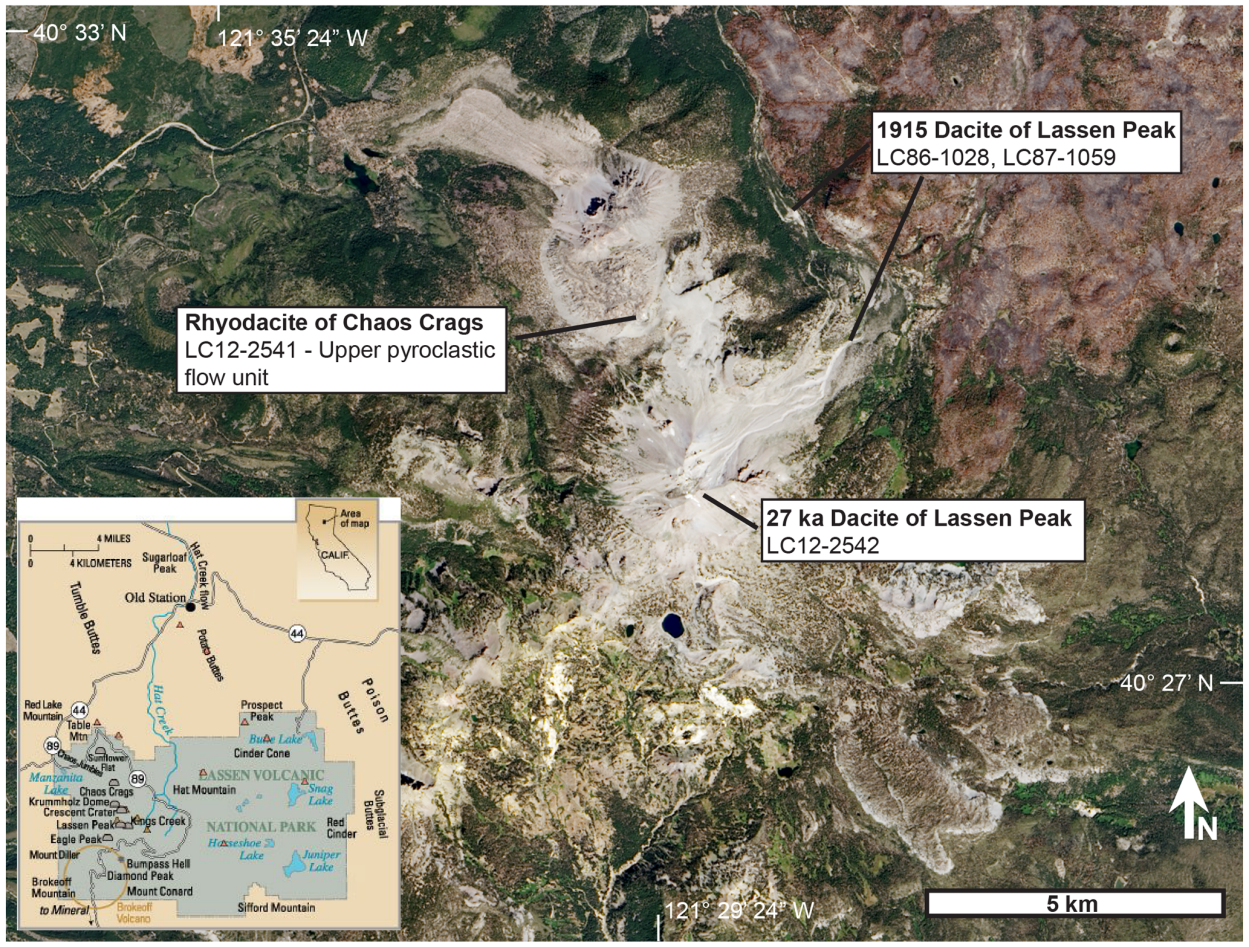
Landsat 8 image of the Lassen Volcanic Center, taken September 2013. Locations for the samples used in this study are marked. Inset map shows the location of the Lassen Volcanic Center along with important volcanic peaks within the LVC (from [58]; USGS Fact Sheet 022-00). Landsat image created by Rob Simmon (Planet Labs, formerly NASA Earth Observatory). Landsat 8 image courtesy of the U.S. Geological Survey.

The Lassen Volcanic Center (LVC), located in northern California (Figure 1), marks the southernmost extent of active volcanism in the Cascade Range. The LVC sits on continental crust that is ∼38±4 km thick [29], of which the uppermost ∼4 km is late Pliocene to Holocene volcanic and intrusive rocks [30]. The bulk of the crustal column is Mesozoic to Paleozoic ultramafic to felsic intrusive bodies and metamorphic rocks related to the Sierra Nevada and Klamath basement terranes. The deeper crust is interpreted to be granulitic and dominated by Mesozoic to late Cenozoic mafic arc intrusive rocks [31].

On a regional scale, Quaternary volcanism in the southernmost Cascade Range has built a broad platform of overlapping and intercalated mafic to intermediate volcanoes. This platform comprises hundreds of coalescing small- to medium-sized volcanoes (typically up to a few km^3^) consisting predominantly of basalt to andesite [28]. We refer to these as regional volcanoes or regional volcanism. Intercalated within the regional volcanic rocks are a few voluminous (as much as a few hundred km^3^) long-lived (0.5–1 m.y.) foci of volcanism called volcanic centers. Volcanic centers contain a diverse assemblage of rock types from basalt to rhyolite, but are dominated by andesite and dacite. Rocks in the volcanic centers are much more complex lithologically than rocks from regional volcanoes, and geochemical and petrographic evidence for magma mixing and recycling of crystals is abundant [32], [33]. Volcanic centers result from a local increase in the amount of basaltic magma intruded into the crust relative to the background calc-alkaline regional volcanism [31]. Increased mafic flux heats the crust and promotes interaction between mafic magma and the crust. A complex, vertically zoned, magmatic system with localized bodies of magma develops in the crust beneath the volcanic center. As the evolution of volcanic centers proceeds, the crustal component becomes increasingly important until, in the later stages, it dominates the magmatic system. Late in the evolution of each volcanic center, an acidic hydrothermal system driven by heat from cooling subvolcanic silicic magma attests to emplacement of an intrusive body. Using oxygen isotopic evidence, [34] estimated upwards of 130 km^3^ of mafic intrusions at the LVC since 600 ka while thermal and mass balance estimates suggest a minimum of ∼200 km^3^ of mafic input since 650 ka [31].

Five volcanic centers younger than about 3.5 Ma are recognized along the Cascade axis in the Lassen area. The LVC is the youngest of these volcanic (Figure 1). Over the last ∼0.825 m.y., the LVC has erupted at least 215 km^3^ of basaltic andesite to rhyolite [35]. The bulk of the erupted material, however, is intermediate in composition (andesite to dacite). The evolution of the LVC is described as 3 major sequences of deposits: (1) Rockland caldera complex ∼825–611 ka; (2) Brokeoff Volcano ∼600–385 ka, and; (3) Lassen Domefield ∼315–0 ka [28]. The largest single eruption of the LVC was the ∼50 km^3^ Rockland Tephra at ∼611 ka [36] as part of the 75 km^3^ Rockland caldera complex. Following this, Brokeoff Volcano erupted ∼100 km^3^ of dominantly andesite.

Activity of the Lassen Domefield commenced at ∼315 ka with the Bumpass sequence and older Twin Lakes sequence. The Bumpass sequence is 30–50 km^3^ of pyroxene-hornblende dacite to rhyodacite erupted as 15 units, mostly on the northern flank of Brokeoff Volcano [28]. These lavas are heterogeneous and have a mineral assemblage that reflects hotter conditions within the magmatic system than the subsequent Eagle Peak sequence rocks [28]. Following the Bumpass sequence was a period of volcanic quiescence in the LVC that lasted from 190 to 93 ka, and probably reflects the reduced rate of mafic volcanism in the Lassen region during the period from 200 to 110 ka [28]. It is unclear whether this lull reflects changes in mantle-derived inputs or tectonic control of ascending magma.

Volcanism in the Lassen Domefield resumed at ∼93 ka shortly after the return of regional volcanism. Subsequent eruptive units are divided into two concurrent groups based on location and composition: the basaltic andesite to andesite Twin Lakes sequence (93 ka to present) and the dacite to rhyodacite Eagle Peak sequence (66 ka to 1 ka), which together added ∼12 km^3^ of new volcanic material. Overall, eruption rates have declined over the last 90 k.y. in the LVC [37].

Most lavas erupted since 93 ka have a mixed/mingled character, and the fundamental difference between the Twin Lakes and Eagle Peaks sequence lithologies is the proportion of basalt in the mixed magma [28]. The Twin Lakes sequence has a strongly disequilibrium mineral assemblage with co-existing quartz and magnesian olivine, produced by mixing of a minimum of 40% basalt with rhyodacite melts [28]. Lavas of the Eagle Peak sequence are coarsely porphyritic with hornblende, biotite, plagioclase and quartz and exhibit a lesser degree of disequilibrium compared to the Twin Lakes sequence [28]. Composite crystals up to 1 cm are common to abundant in most Eagle Peak lavas [28]. They also typically contain from a few to about 20% quenched mafic inclusions formed by hybridization of basalt with rhyodacite [37], [38]. Disaggregation of inclusions in Eagle Peak units contributed crystals and liquid to the host magmas. The bulk composition and phenocryst assemblage in each Eagle Peak unit is the same, whereas the suite of quenched mafic inclusions in each eruptive unit is unique.

## Samples

Four samples from three of the youngest eruptive events at the LVC were analyzed in this study (Figure 1): two from the Eagle Peak sequence and one from the Twin Lakes sequence. The Eagle Peak sequence samples were the 27±1 ka dacite of Lassen Peak (∼2.07 km^3^) and the 1.1 ka rhyodacite of Chaos Crags (1.19 km^3^). The older sample, dacite of Lassen Peak (LC12-2542) was collected from the lithic pyroclastic-flow deposit from partial collapse of the dome (unit pfl in [28]) and is compositionally equivalent to LC83-392 [39]. It is a porphyritic hornblende-biotite rhyodacite (68.1 wt% SiO_2_) with 30% phenocrysts (plagioclase>quartz>biotite>hornblende with sparse oxides, augite and trace olivine). Olivine, augite, and calcic plagioclase-bearing mafic inclusions, fragments of inclusions, and crystals derived from disaggregated inclusions are abundant. The younger sample, rhyodacite of Chaos Crags (LC12-2541), was collected from the upper pyroclastic flow (unit pc in [28]) and is compositionally equivalent to LC84-419 [39]. It is a porphyritic hornblende-biotite rhyodacite (69.4 wt% SiO_2_) with 35% phenocrysts (plagioclase>biotite>hornblende>quartz with sparse oxides). Olivine, augite, and calcic plagioclase-bearing mafic inclusions, fragments of inclusions, and crystals were derived from disaggregated inclusions are sparse.

Two samples from the dacite erupted from Lassen Peak in 1915 (0.03 km^3^) were examined. LC86-1028 is black dacite lava (64.9 wt % SiO_2_) collected from the May 19–20, 1915 mudflow and was likely part of the lava dome erupted between May 14 and May 19 (unit d19 in [40]). LC87-1059 is gray dacite (64.4 wt% SiO_2_) collected from a larger block of unbanded light gray pumice from the late May 22, 1915 mudflow (unit wv22 in [40]). Both samples are porphyritic biotite-hornblende dacite containing about 30% phenocrysts of plagioclase>biotite = hornblende>quartz, and are compositionally equivalent. Olivine-bearing mafic inclusions, fragments of inclusions, and crystals derived from disaggregated inclusions are abundant (see [39] for full descriptions of the dacites and inclusions). In all samples included in this study, zircon is found in thin section, both in the groundmass (or glass) or included within other phases such as biotite and plagioclase feldspar.

## Methods

**Figure 2:**
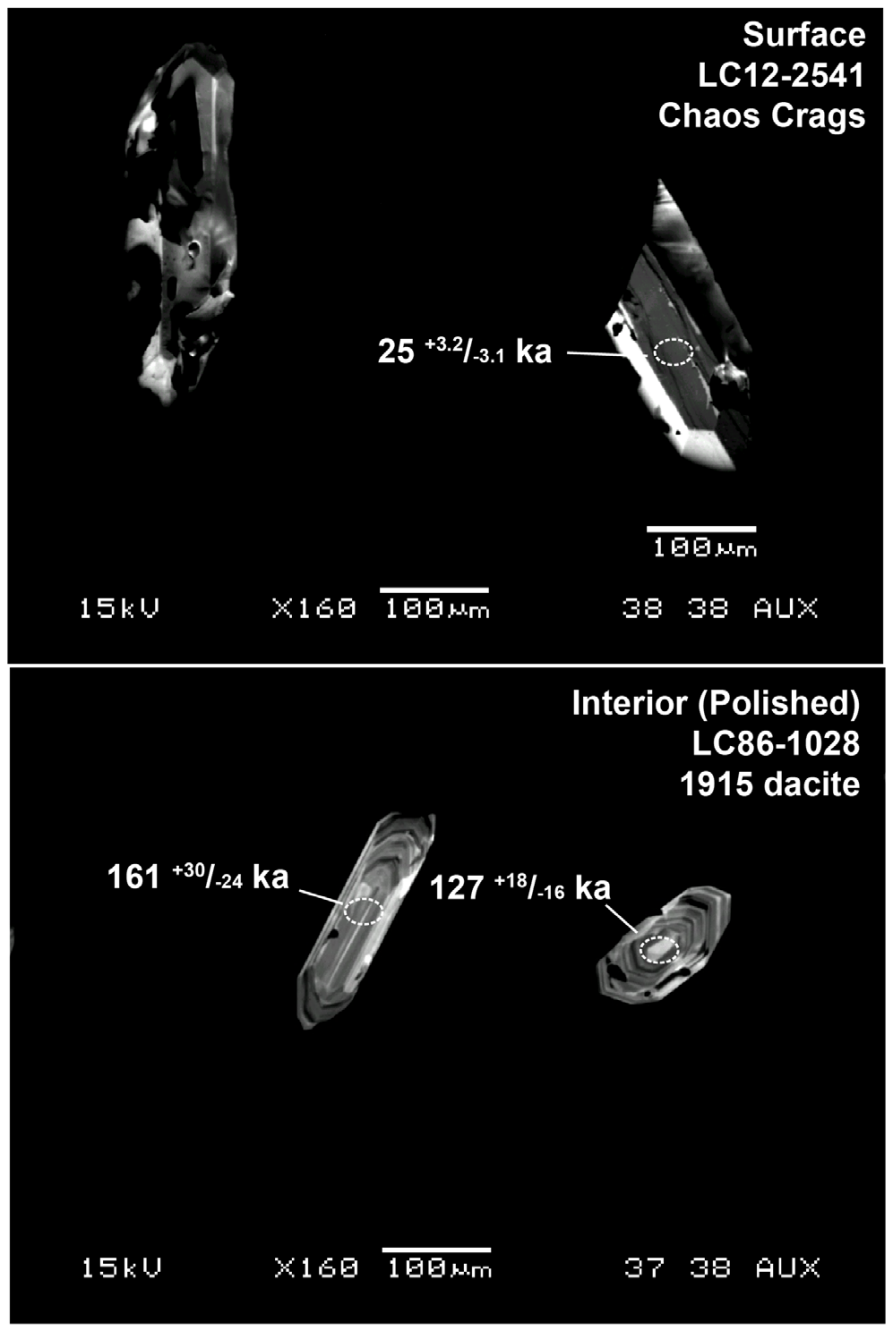
Cathodoluminescent images of zircon analyzed via SHRIMP-RG in this study. Top panel shows zircon used in surface analysis, mounted in indium. The bottom panel shows two zircon used in polished interior analysis, mounted in epoxy. All ages are reported with 1σ errors.

All samples (LC12-2542, LC12-2541, LC86-1028 and LC87-1059) in this study collected under the auspices of the US Geological Survey (by Clynne) with permission from the National Park Service. All materials from the samples were destroyed during the sample preparation process. The samples were processed using standard mineral separation techniques to extract zircon (see [41]). Zircon from each sample were mounted in epoxy, polished and imaged via cathodoluminiscence at the Stanford/USGS SUMAC facility to examine the zoning and presence of inclusions within the grains (Figure 2). Additional grains from each sample were mounted in indium metal in order to analyze flat outer surfaces of grains. These were also imaged by cathodoluminiscence to examine the surface zoning and look for relatively glass-free surfaces on the zircon (Figure 2). Most zircon were 50–200 µm in length and 30–100 µm in width.

Selected zircon (n = 80) were analyzed via SHRIMP-RG at the Stanford/USGS SUMAC for ^238^U-^230^Th isotopic composition (Appendix S1). A total of 105 zircon were analyzed for trace elements (following [4], [42], [43]; see Methods S1 for full methodology), however only 84 produced data (Appendix S1) that did not suggest contamination by glass inclusions in the analysis, such as anomalously high K and Na content in zircon, are included. Some of these zircon do not have corresponding U-Th ages. Only Hf, Yb, U, Th and Gd concentrations and Eu/Eu* are presented here, with additional analyses of Li, Be, B, F, Na, Al, P, Ca, Sc, ^48^Ti, ^49^Ti, Fe, Y, Nb, La, Ce, Nd, Sm, Eu, Ho, Tb, Dy, Er, Tm, Lu and Pb and analytical uncertainties of the trace element measurements presented in Appendix S1. Interiors and surfaces of grains were analyzed during analytical sessions in July 2011, November 2012 and August 2013 (Appendix S1).

[44] reported initial whole rock values for (^238^U/^232^Th) and (^230^Th/^232^Th) in young LVC rocks. These vary from 0.988±0.031 to 1.095±0.046 and from 1.035±0.035 to 1.114±0.044, respectively. Values reported in [45] are similar. Selecting an initial value to calculate zircon ages is problematic, as values for the liquid in which the zircon grew are not known. With the limited range of values from [44], we choose a value that is a representative average of the LVC magmatic system over the past 100,000 years (1.05 for (^238^U/^232^Th) and 1.08 for (^230^Th/^232^Th). If the range in initial (^238^U/^232^Th) and (^230^Th/^232^Th) is allowed to vary between reported values, the average change in calculated model age is +4.9/−5.9% for (^238^U/^232^Th) and +3.9/−5.9% for (^230^Th/^232^Th), respectively. Thus, choice of initial (^238^U/^232^Th) and (^230^Th/^232^Th) do not significantly affect the calculated ages or the interpretations herein.

Ti-in-zircon temperatures were calculated using [46], assuming a(Si) of 1.0 and a(Ti) of 0.6 (Appendix S1). The presence of quartz in these samples supports an a(Si) of 1.0, especially in potentially more fractionated crystal mush. For zircon crystals included in other phases, it is assumed that the magma in which the major phase formed reflects the magma in which the zircon formed, so a(Si) = 1 is still applicable. In all cases, a variation of a(Si) of ±0.05 would change calculated temperatures by ±5°C. For most calc-alkaline arc magmas, a(Ti) is between 0.5–0.8 [47]. We chose 0.6 to reflect the a(Ti) from Mount St. Helens [47] as the closest analog for the LVC. Errors in activity estimates of 0.2 would change calculated temperatures by 30°C [43]; however these will shift all temperatures systematically and would not significantly affect the interpretations. Analytical errors on the ^48^Ti measurements, relative to the MADDER zircon standard, were 3.8% (1σ), leading to average errors based on analysis on all temperatures of ±5°C (1σ).

## Data

### Zircon ^238^U-^230^Th ages

**Figure 3:**
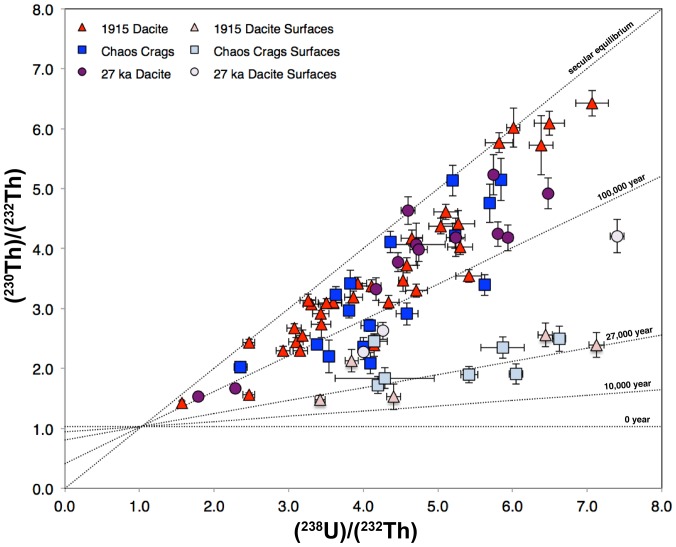
^238^U-^230^Th isochron diagram showing interior (dark) and surface (light) analyses from the three eruptive units analyzed from the LVC. Error bars shown are 1σ, with reference isochrons (10, 27, 100 k.y., along with the equiline). See Appendix S1 for full age and other compositional data.

**Figure 4:**
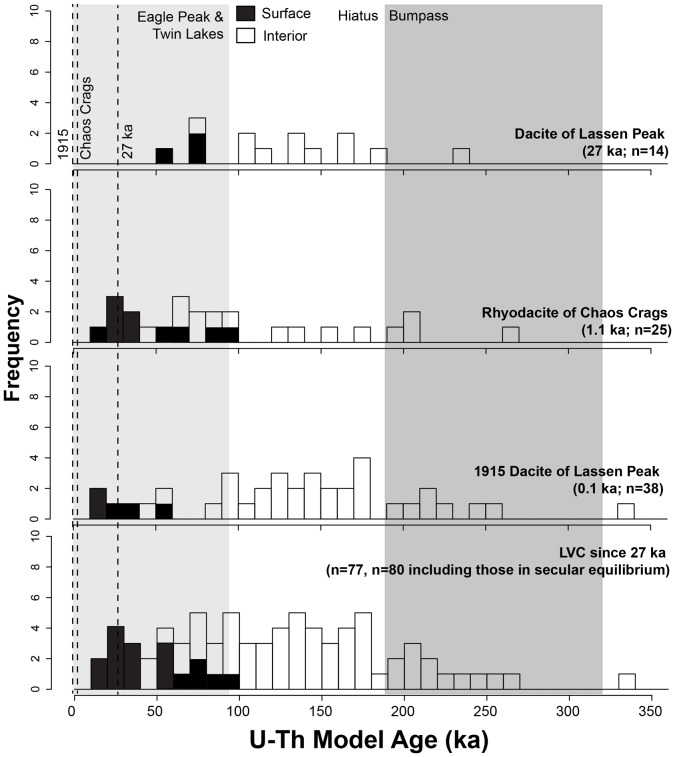
Zircon ^238^U-^230^Th model age distribution histograms of zircon from the three eruptive units analyzed from the LVC: ∼27 ka dacite of Lassen Peak (top), ∼1.1 ka rhyodacite of Chaos Crags (top middle) and 1915 dacite of Lassen Peak (bottom middle). The bottom histogram compiles all the zircon ages from across all three units. Parts of the histograms in black represent the surface (rim) analyses of zircon, while white are polished interior analyses. See Appendix S1 for full ages and errors.

^238^U-^230^Th model ages of analyzed zircon range from ∼15.6 ka to secular equilibrium (≥350 k.y.; Figure 3, Appendix S1). A majority of zircon analyzed fall between 60–200 ka, regardless of the age of the host rock, with fewer zircon to 280 ka (Figure 4). Rare zircon are within error of secular equilibrium (4 of 80) and are likely either inherited from older volcanic rocks or continental crust. Little-to-no difference in zircon ages was observed between the black and grey dacite samples from the 1915 dacite (LC86-1028 and LC87-1059; Appendix S1); thus they are considered the same sample for the remainder of this study. When comparing between the three eruptive units (Figure 1), the overall zircon age populations are similar, although the rhyodacite of Chaos Crags and dacite of Lassen Peak have a greater proportion of 60–110 ka zircon versus the 1915 dacite where more zircon fall between 100–180 ka. None of the zircon analyzed, both as surfaces and interiors, were within error of eruption age of the host lava (Figure 4). The youngest interior age found in each eruptive unit was 44^+4.7^/_−4.5_ ka (rhyodacite of Chaos Crags), 45^+13.8^/_−12.3_ ka (1915 dacite) and 71^+6.9^/_−6.5_ ka (dacite of Lassen Peak).

Surface analyses of zircon (18 of 80; Appendix S1) produced ages younger than any interior analysis (Figure 3). However, the surface population was not distinct, ranging in age from 15.6–96 ka. Within the individual eruptive units, there were no patterns in the distribution of surface ages. The 1915 dacite and rhyodacite of Chaos Crags yield the youngest surface ages that are within error of each other (15.6^+8.4^/_−7.9_ and 19.8^+4.6^/_−4.4_ ka respectively; Appendix S1). The youngest surface age from the dacite of Lassen Peak was 57^+6.0^/_−5.7_ ka, outside of error envelope of the eruption age. Thus, no surface age overlaps with the eruptive age of the rock units.

### Zircon trace element compositions and Ti-in-zircon temperatures

**Figure 5:**
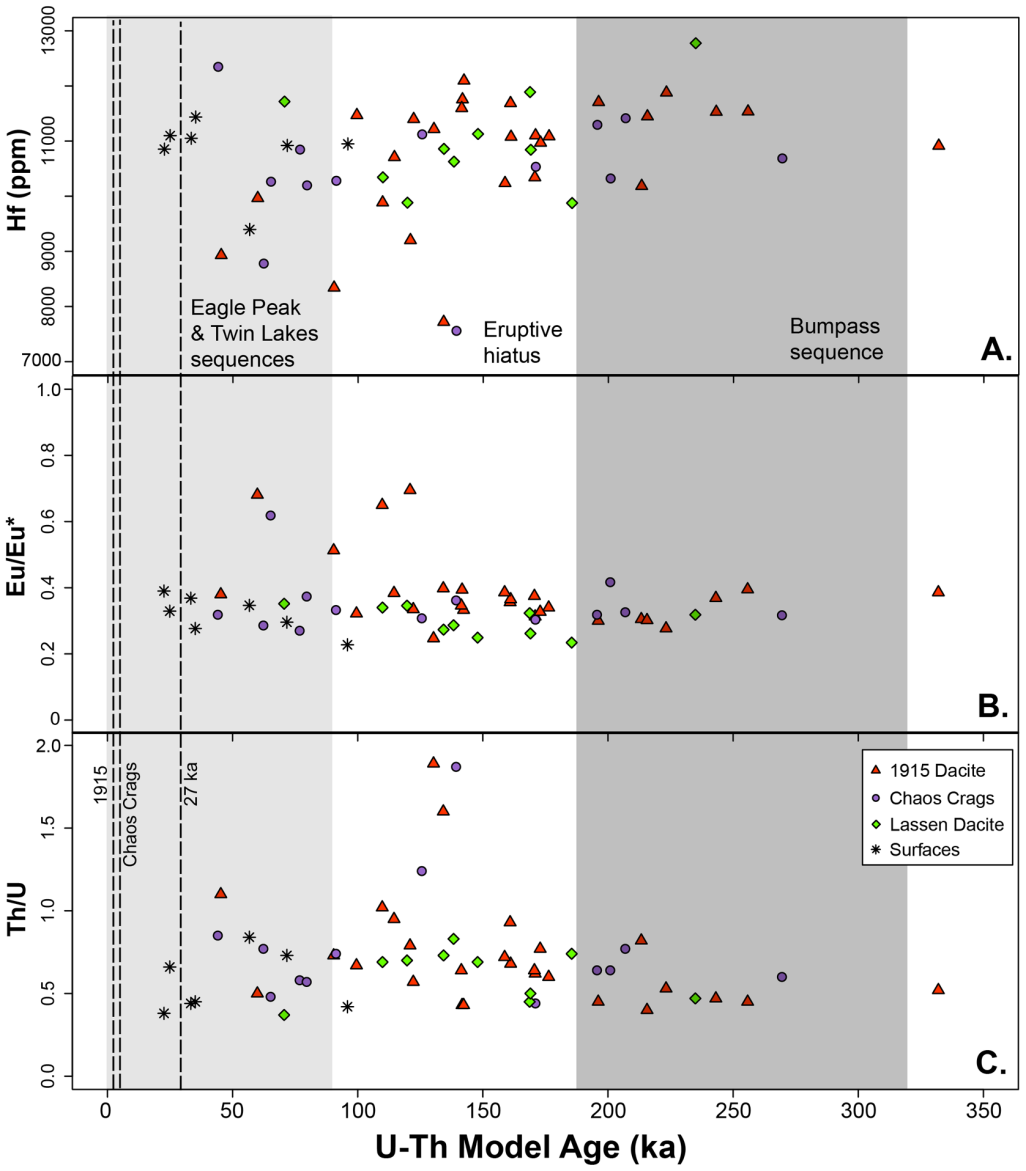
^238^U-^230^Th model ages versus selected trace element abundances or ratios. Bumpass sequence (350-190 ka; dark grey) and the Eagle Peak and Twin Lakes sequences (<90 ka; light grey; both from [37]) are marked on the figure, along with the eruptions from which the zircon were sampled (dashed lines). The eruptive hiatus (190-90 ka) is marked in white. From top to bottom, (a) Hf (ppm) of zircon surfaces and interiors; (B) Eu/Eu* of zircon surfaces and interiors; (c) Th/U of zircon surfaces and interiors.

**Figure 6:**
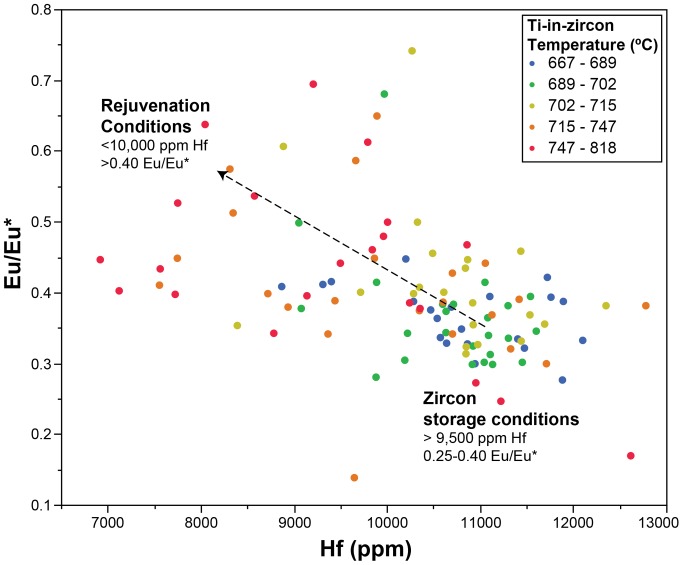
Hf versus Eu/Eu* for LVC zircon, color-coded for Ti-in-Zr temperatures. At temperatures greater than approximately 730°C (Hf <9,500 ppm), Eu/Eu* varies across a much wider range (0.4–0.75). This represents the zircon growth during the down-temperature path after a rejuvenation event while those with Eu/Eu* between 0.2–0.4 and Hf >9,000 ppm represent growth in the crystallizing mush in baseline zircon storage conditions.

**Figure 7:**
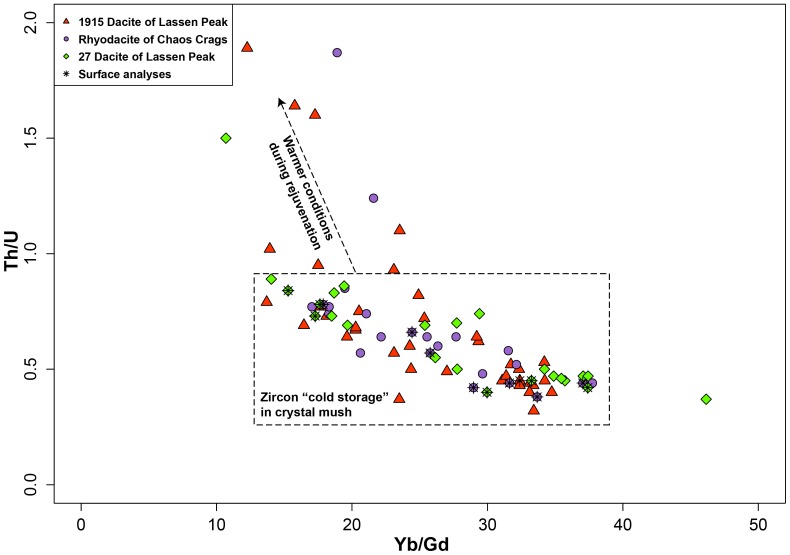
Yb/Gd versus Th/U for LVC zircon. Crystal mush “cold” storage conditions are marked with a dashed box while the dashed line with the arrows marks the path to warmer conditions during rejuvenation events. Th/U should increase while Yb/Gd decreases as greater basaltic input heats the crystal mush [41], [51].

Overall, Hf concentration in the analyzed zircon ranges from 6915 to 12780 ppm (Figure 5a). A majority of the zircon have >10,000 ppm Hf (Figure 5a). In all three samples, Hf abundance in surface analyses of zircon overlaps with Hf abundance in cores; however all surfaces have >9,000 ppm Hf. In most zircon analyzed, Eu/Eu* is limited to 0.25 to 0.4 (Figure 5b) while a minority of zircon younger than 130 ka have higher Eu/Eu* (0.4 to 0.75; Figure 5b), which correlate with <10,000 ppm Hf (Figure 6). Th/U ratios fall between 0.4 and 1 for most of the zircon analyzed (Figure 5c). However, a small population have Th/U >1, all of which have model ages between ∼120–130 ka. Yb/Gd clusters between 12–40 (Figure 7), with one zircon at 47. A compilation of all trace element analyses is provided in Appendix S1.

**Figure 8:**
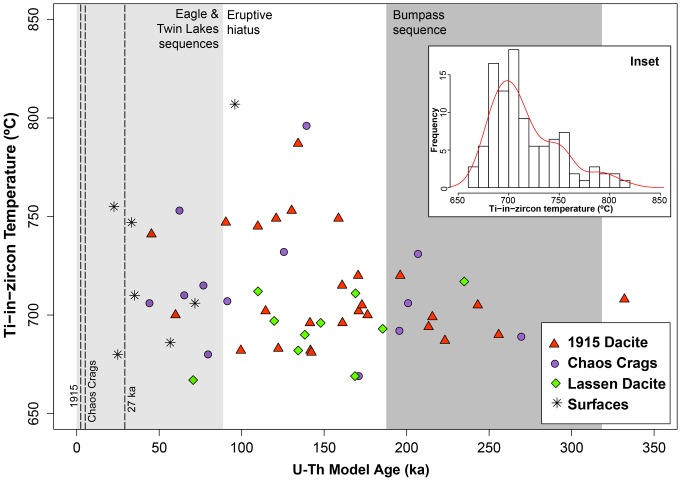
U-Th model age versus Ti-in-zircon temperatures from all three eruptive units analyzed. A majority of zircon analyzed fall between 680–725°C, while a subpopulation is warmer, with a range between 730–820°C. The three eruptive sequences and three eruptions sampled are marked in the same fashion as Figure 5. *Figure 8* *Inset*. Histogram of Ti-in-zircon temperatures for the LVC with a probability density function for the data (red line), showing the main population centered around 700°C and a smaller peak of higher-T zircon above 735°C (small shoulder to right of main peak).

When the Ti-in-zircon thermometer of [46] is applied (Figure 8), zircon crystallization temperatures range from 667–818°C, with a majority of the zircon falling between 670–725°C, with a secondary peak between 735–820°C (Figure 8 inset; Appendix S1). There is little variation in the temperature distributions across the three units, surface analyses and the total LVC zircon population (Table 1). These temperature show a correlation with Hf (ppm) and Eu/Eu* (Figure 6), where lower-T zircon have higher Hf and lower Eu/Eu* and higher-T zircon have lower Hf and higher Eu/Eu*. Glass analyses from Chaos Crags Dome A [48] yield zircon saturation temperatures of 709–769°C (using methods from [49]), assuming a range of Zr concentrations between 100–180 ppm (Table 2), with 727°C for our chosen Zr concentration (120 ppm) based on whole rock composition. Additionally, representative silicic magmas erupted during the Bumpass sequence (the rhyodacite of Mt. Conard), the rhyodacite of Chaos Crags (Eagle Peak sequence) and the 1915 dacite of Lassen Peak (Twin Lakes sequence) yield maximum zircon saturation temperatures (∼715°C, ∼705°C and ∼695°C respectively, using methods from [49]; Table 2). All of these zircon saturation temperatures are below the maximum inferred magmatic storage temperatures of 760–775°C of the remobilized Chaos Crags magma [48] that was likely heated prior to eruption to as high as 800°C (at 5 kb) based on Ti-in-quartz analyses of quartz rims [50] and ∼820°C based on Fe-Ti oxides [48].

**Table 1:** Ti-in-zircon temperatures and ^48^Ti (ppm) for each unit, surfaces and complete LVC zircon.

Unit	Number of Analyses	^48^Ti range (ppm)	Temperature Range (°C)	Mean Temperature (°C)	Median Temperature (°C)
1915 Dacite of Lassen Peak	36	2.8–9.1	678–787	718	707
Rhyodacite of Chaos Crags	16	2.5–10.0	669–796	714	709
27 ka Dacite of Lassen Peak	18	2.4–9.7	667–793	706	697
Surfaces	14	2.8–12.3	679–818	719	700
All LVC since 27 ka	84	2.4–12.2	667–818	715	706

Ti-in-zircon temperatures and ^48^Ti (ppm) for each unit, surfaces and complete LVC zircon. All temperatures calculated using a(Si) = 1.0, a(Ti) = 0.6 with methods from [46].

**Table 2:** Composition, age, volume and T_Zr_ (calculated using [49]) for selected lavas of the Lassen Volcanic Center.

Unit	Sequence	SiO_2_ (wt%)	Zr (ppm)	Eruption Age (ka)	Volume (km^3^)	T_Zr_ (C)#
1915 Dacite of Lassen Peak	Twin Lakes	65.5–68.5*	105–137	0.1	0.03	666–685 (675)
Rhyodacite of Chaos Crags	Eagle Peak	67.2–70.2	121–140	1.1	1.19	688–716 (705)
27 ka Dacite of Lassen Peak	Eagle Peak	62.9–70.2	109–141	27	2.07	674–713 (695)
Rhyodacite of Mt. Conard	Bumpass	69.4	199	298	n/a	742
Glass from the rhyodacite of Chaos Crags^2^	Eagle Peak	80.3	100–180	1.1	1.19	727 (for 120 ppm Zr)

All compositional and age data from [28], [37].

* does not include dark bands within banded pumice that are not relevant for calculating the T_Zr_ for the 1915 dacite.

# Range of temperatures derived from multiple compositions and number in parentheses is the selected value for the unit.

2 Glass analysis from [48].

## Discussion

Zircon from the most recent three silicic eruptions from the Lassen Volcanic Center reflect multiple samplings of the crystal mush that has developed since the Bumpass sequence (190–315 ka). This mush is thought to have been remobilized across the recent history of the LVC since at least ∼90 ka. Hafnium content of zircon has been shown to be a proxy for the extent of crystallization in a silicic magma [8], [43] while Eu/Eu* tracks the extent of plagioclase fractionation [41]. The systematic variation in Hf and Eu/Eu* compositions of the zircon reflects both crystallization of zircon within the cooling crystal mush (high Hf, low Eu/Eu*) and the down-temperature crystallization of zircon after a remobilization (rejuvenation) event (low Hf, high Eu/Eu*).

### Zircon age spectrum

The first observation that can be made of the zircon age spectrum from the three most recent silicic eruptions of the LVC (Figure 4) is that none of the zircon analyses – interiors or surfaces – are within error of the ages of eruptions from which they were sampled (Figure 3). This pattern of antecrystic (rather than autocrystic) zircon is a common occurrence at continental arc settings (e.g., [4], [8], [51]). The small difference in the age distribution between units in the LVC could reflect the difference in thermal (basaltic) input, as the hotter magma of the 1915 dacite will cause more rapid zircon dissolution. Both the cores and surfaces of the zircon overlap with eruptive activity within the LVC. However, a majority of the zircon analyzed crystallized during a period of eruptive quiescence between 190 and 90 ka (Figure 5). The surface ages are, on average, younger than the cores, but they are still much older (by at least 10^4^ years) than their host eruption (Figure 4).

This age spectrum supports a model where zircon are sampled from crystal mush that underlies the central portion of the LVC. This mush has a low portion of melt during much of the recent history of the LVC, as seismic studies of the subsurface at the LVC suggest [37]. Although Chaos Crags and Lassen Peak are separated by 5 kilometers and 27,000 years, zircon age spectra of the three silicic eruptions are similar (Figure 4). This, along with bulk composition and phenocryst assemblages [28], [39], support the idea that the each eruption is sampling the same syn- and post- Bumpass sequence crystal mush.

### Crystal mush storage conditions

Zircon trace element compositions can provide information on the conditions that the crystal mush is stored and remobilized during a new injection of basalt as individual zircon and zircon populations can be used to look at changing conditions in the magmatic system. Used in tandem with the Ti-in-zircon geothermometer [46] and U-Th ages, the thermal variations across the history of the crystal mush can be assessed.

In the LVC, most zircon fall within a restricted range of trace element abundances and ratios (Figures 5–8), which can be used to define the baseline conditions of the crystal mush: 0.25–0.4 Eu/Eu*, 9,500–13,000 ppm Hf and Yb/Gd 12–40. These compositions reflect crystallization of zircon at conditions of ∼670–725°C, based on Ti-in-zircon thermometry (Figures 7 and 8). This is cooler than the inferred maximum pre-eruption storage conditions for the rhyodacite of Chaos Crags [48] but overlaps with calculated zircon saturation temperatures for the 1915 dacite, rhyodacite of Chaos Crags and the silicic magmas of the Bumpass sequence, along with the glass of the rhyodacite of Chaos Crags (Table 2). This suggests that the bulk of the subvolcanic crystal mush is stored at conditions just below zircon saturation to allow for growth of new zircon crystals and layers of zircon on pre-existing crystals. When considering Eu/Eu*, Yb/Gd and Th/U, the conditions of the mush strongly promote plagioclase and zircon crystallization, driving all three ratios to respectively lower values as the temperature of the mush decreases [41], [51].

### Remobilization events captured in zircon

**Figure 9:**
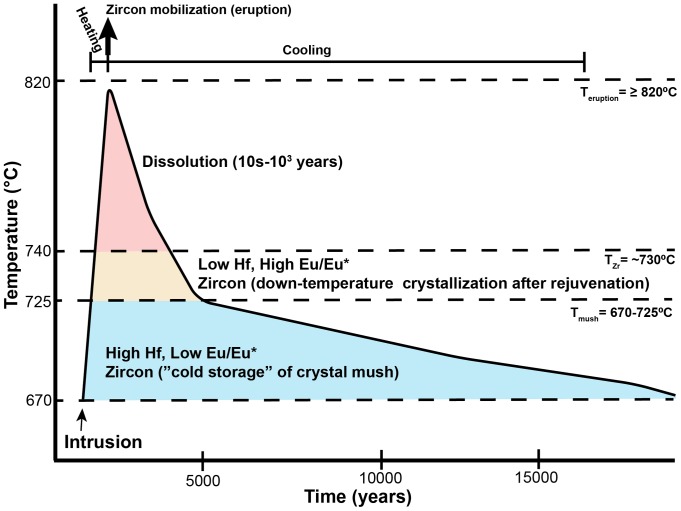
Schematic model of a rejuvenation event that leads to an eruption at the LVC, assuming a bulk composition similar to the Dome A of Chaos Crags. The dark curve marks the changing thermal conditions within a zone of rejuvenation after a basaltic intrusion. The light blue area represents baseline zircon storage conditions in the crystal mush based on Ti-in-zircon temperatures (Figure 8), the zone of “cold storage” of crystals. The orange zone represents the area where zircon will crystallize during the down-temperature path of the rejuvenation event. The red area represents the thermal conditions above the highest zircon saturation temperatures calculated from glass composition in the rhyodacite of Chaos Crags [48] with 120 ppm Zr (Table 2), which is assumed to be close to the crystal mush liquid composition. In this zone, zircon will dissolve in 10^1^–10^3^ years. The solid bars at the top represent the short timescale of heating (years to decades) and longer timescale of cooling (10^3^–10^4^ to return to ∼670°C) from the rejuvenation. T_eruption_ (820°C) is the maximum temperature the magma reaches during rejuvenation, recorded in Ti-in-quartz analyses of quartz rims [50]. The temperature of the cooling mush is based on Ti-in-zircon temperatures derived from the zircon analyzed. The two populations of zircon, from baseline crystallization in the mush and down-temperature cooling from previous rejuvenations mix as the mush moves from locked in “cold storage” to “unlocked” during new heating events. The ages presented are based on zircon dissolution models [56] and are highly dependent on the composition of the host magma and thermal conditions, but provide maximum limits to the duration of rejuvenation heating.

Not all zircon analyzed from these three most recent silicic eruptions in the LVC fall within the crystal mush baseline composition. A subpopulation shows characteristics that suggest they crystallized at slightly warmer (>735°C; Figure 8), where the heat was provided by the rejuvenation of the mush due to basaltic intrusion. These zircon likely capture a portion of the down-temperature history of a localized remobilization event within the LVC mush, prompted by an injection of basaltic magma (Figure 9). Some of these events lead to an eruption, as suggested for the 1915 dacite of Lassen Peak [38]. However, some of these remobilization events never manifest as eruptions. Instead, they help in redistributing crystals through the mush by carrying small (<0.05 mm) zircon grains in the viscous interstitial rhyolite liquid generated during the rejuvenation [52]. Repeated rejuvenation would then create a uniform crystal age distribution that can be sampled by eruptions at different locations and times in the recent history of the LVC (Figure 4) within zones of preferential crystal transport [21]. This is similar to patterns of plagioclase recycling within andesitic systems such as Soufriere Hills [23], [24], where diverse plagioclase crystal populations, distinguished by their composition, are remobilized during each new magmatic intrusion. This creates an amalgam of crystal populations that, based on Sr and Ba diffusion in plagioclase, likely mixed in ∼10–1200 years [24]. Remobilization of crystals has been observed in the plutonic record in large-scale systems such as the Tuolumne Intrusive Complex [21], [22], the Spirit Mountain Batholith [43], Crater Lake [53] and the Vinalhaven Pluton [19], amongst others. The LVC is likely a much smaller magmatic system than any of these systems, but all show the importance of localized remobilization of crystals in response to thermal rejuvenation.

The crystallization of cooler-T zircon in the baseline crystal mush conditions (670–725°C) is analogous to the model of “cold storage” of crystals suggested by [54]. In the LVC, the occurrence of zircon with <10,000 ppm Hf, >0.4 Eu/Eu* and Ti-in-zircon temperatures >730°C represent the conditions of down-temperature cooling after a localized remobilization event (Figure 6). None of these zircon are older than ∼130 ka and most crystallized after the hiatus and the initial eruptions of Twin Lakes and Eagle Peak sequences (<95 ka; Figures 5–8). In these localized remobilization events, basaltic input heats the systems to a point where the crystal mush no longer behaves rigidly and instead becomes eruptible, likely when liquid content of the mush approaches 60% [55]. After the rejuvenation and remobilization, the system cools back to baseline conditions. Thus, the warmer-T zircon represent the remnants of rejuvenation of crystal mush from “cold storage” to eruptibility.

Timescales for these localized remobilization events can be estimated by considering the zircon saturation state of the magma prior to eruption. All the erupted magmas in this study are zircon undersaturated (Table 2), so zircon should be actively dissolving. Although many zircon are preserved in other mineral phases such as plagioclase, hornblende, quartz and biotite, there are zircon present in the groundmass. The zircon saturation temperature for glass in the rhyodacite of Chaos Crags with 120 ppm Zr is ∼727°C, which is at the transition between the baseline storage temperature of the crystal mush, so even small thermal perturbations will push the liquid within the mush out of zircon saturation (without an accompanying change in composition, as provided by addition of basalt).

Using a representative glass composition from the rhyodacite of Chaos Crags ([48]; Table 2) as an analog for the liquid composition of the crystal mush, estimates can be made on rates of dissolution of zircon at different thermal conditions (using methods from [56]). If the magma is heated to 820°C (M = 1.26, U = 51 ppm) as suggested by Fe-Ti oxide temperatures from the rhyodacite of Chaos Crags [48], a 60-µm diameter zircon will dissolve in ∼8,300 years and a 20-µm diameter zircon with dissolve in ∼933 years. If heated to 900°C (M = 1.26, U = 308 ppm) as suggested for the mixed 1915 dacite of Lassen Peak, the same zircon will dissolve in less than 235 and 26 years, respectively. This offers constraints on the timescale for the thermal perturbation caused by localized remobilization. These remobilization events are likely triggered by small injections of magma, with volumes of basaltic input less than 1 km^3^ based on mass balance and petrographic evidence from the 1915 dacite of Lassen Peak and rhyodacite of Chaos Crags [37]. If heating within the zone of remobilization lasted for more than a few tens to a few thousand years, depending on the temperature of rejuvenation, then no zircon should have survived within the groundmass of the erupted LVC lavas. Their presence means that the total history of a remobilization event lasts, at most, a few thousand years and that much of the petrologic history recorded in these crystals are from the down-temperature growth after eruption (or disruption from equilibrium conditions). These timescales are consistent with those calculated from major phases in the Bishop Tuff [16] and Soufriere Hills [24].

Mixing events prior to an eruption were likely brief [38], at most a few years to decades before the eventual eruption based on mineral reactions observed in the erupted lava. This means that the localized zone of remobilization would experience rapid heating through convective mixing and conduction from the injecting basalt, followed by a slower cooling after the eruption or end of basaltic injection. It is in this down-temperature path after rejuvenation that the higher temperature zircon are thought to crystallize (Figure 9), as the localized rejuvenation zone cools below zircon saturation on its way to the ambient conditions of the crystal mush. These zircon are rarer than the lower-temperature zircon as they only crystallize in the remobilization zones during cooling, unlike the baseline zircon that crystallize throughout the history and footprint of the crystal mush.

Subsequent remobilization and production of newly eruptible magma during rejuvenation through preferred pathways, as repeated eruptions from the Lassen Peak and Chaos Crags vents attest, allow for mixing of the crystal cargos (baseline crystal mush and rejuvenated crystal mush). Also, the abundant petrographic evidence of zircon included in other crystals (along with zircon clearly in contact with melt) in these LVC lavas suggests that major phases are also recycled repeatedly throughout the history of the LVC mush. However, the lack of zircon surfaces or interiors with ages less than 17 ka suggest that the preferred pathways of rejuvenation are hot enough to prevent new zircon growth or dissolve any very small zircon crystals that nucleated since 17 ka, likely due to higher proportions of basaltic interaction as exhibited in the 1915 dacite [38].

## Conclusions

The two most recent eruptions at Lassen Peak and the series of domes at Chaos Crags appear to represent magma drawn from rejuvenation pathways within the cooling Bumpass sequence magma body. Rejuvenation of semi-solidified crystal intrusive bodies has been suggested at many silicic magmatic systems [4], [9], [20], [27]. This study shows the importance of crystal recycling in long-lived magmatic systems [7], [8], [18], [57], even when the main body may be approaching solidus conditions. In a long-lived magmatic system, periods of quiescence allow for the formation of crystal mushes. However, even periods of relatively lower eruptive output can produce local rejuvenation of the cooling pluton that can last decades to millennia. This is likely the dominant mode of magmatic evolution during the waning stages of a long-lived arc magmatic system. Abundant crystal exchange (both of major and trace phases) and mixing occur within these preferred remobilization pathways during rejuvenation with little disturbance of the larger cooling pluton. In this way, a dominantly homogenous appearance can be achieved with localized areas of complex magmatic structures and crystal movement that capture the last stages of active magmatism within a cooling plutonic body. These data also suggest that each rejuvenation event lasts, at maximum, tens to thousands of years, with much of that history in the down-temperature path after basaltic injection, so that much of the petrologic history recorded in crystals reflects events after and between eruptions. Thus, a waning magmatic system can still produce silicic eruptions through small volume intrusions of basalt that result in rejuvenation and remobilization of a crystal mush.

## Supporting Information

Appendix S1 **Zircon trace element abundances with analytical uncertainties and U-Th model ages and errors, derived from SHRIMP-RG analyses of selected zircon from the most recent silicic eruptions in the Lassen Volcanic Center.**
(XLSX)

Methods S1 **Full methods for trace element analyses of zircon via SHRIMP-RG.**
(DOCX)
