# Supplementary material for: Localized Rejuvenation of a Crystal Mush Recorded in Zircon Temporal and Compositional Variation at the Lassen Volcanic Center, Northern California
Source: PLoS One. 2014 Dec 3;9(12):e113157. doi: 10.1371/journal.pone.0113157 (PMC4254462; doi:10.1371/journal.pone.0113157)
Supplement: Methods S1 — Full methods for trace element analyses of zircon via SHRIMP-RG. (DOCX) [file pone.0113157.s002.docx]

**Analytical SHRIMP-RG Methods**

Modified from Methods by Matthew Coble (Stanford University)

Zircon trace element analyses were conducted on the SHRIMP-RG (reverse geometry) ion microprobe co-operated by U.S. Geological Survey and Stanford University in the SUMAC facility at Stanford University between 07/27/2011, 11/30/2012-12/01/2012, and 08/05/2013 (mounts EKLP-1, EKLP-3, and EKLP-5, respectively). For mount EKLP-1, zircon grains, concentrated by standard heavy mineral separation processes and hand selected for final purity, were mounted on double-sided tape on a glass slide in ca. 1 x 6 mm rows, cast in a 25 mm diameter by 4 mm thick epoxy disc, ground and polished to a 1 micron finish. For mounts EKLP-3 and EKLP-5, zircon was hand selected from heavy mineral concentrates and mounted on glass slides coated with a thin (<10 microns) film of vacuum grease. This grease allows the grains to be easily manipulated and arranges in ca. 1 x 6 mm rows with the flat euhedral zircon surfaces (m-face) oriented down, against the glass. Oriented zircons grains were pressed into a 25 mm diameter pre-polished indium, exposing flat non-polished zircon surfaces parallel to the mount surface. All grains were imaged with reflected light on a petrographic microscope. Polished grains on EKLP-1 were also imaged with cathodoluminescence on a JEOL 5600 SEM to identify internal structure, inclusions and physical defects. The mounted grains were washed with a 1N HCl solution and thoroughly rinsed in distilled water, dried in a vacuum oven. The sample surface was coated with and ~100Å Au was applied and inspected to ensure uniformity and conductivity before loading into the pre-load instrument chamber. The mounts were stored at high pressure (10^-7^ torr) for several hours before being moved into the source chamber of the SHRIMP-RG to minimize degassing of the epoxy and isobaric hydride interferences.

For trace element analyses of zircon measured on the SHRIMP-RG, secondary ions are sputtered from the target spot using an O_2_^-^ primary ion beam with an intensity varying from 1.4 to 2.4 nA. The primary ion spot had a diameter between 20-30 microns and a depth of ~1.5 micron for the analyses performed in this study. Before every analysis, the sample surface is cleaned by rastering the primary beam for 60 seconds, and the primary and secondary beams are auto-tuned to maximize transmission. The acquisition routine includes analysis of ^7^Li^+^, ^9^Be^+^, ^11^B^+^, ^19^F^+^, ^23^Na^+^, ^27^Al^+^, ^30^Si^+^, ^31^P^+^, ^39^K^+^, ^40^Ca^+^, ^45^Sc^+^, ^48^Ti^+^, ^49^Ti^+^, ^56^Fe^+^, ^89^Y^+^, ^93^Nb^+^, ^92^Zr^1^H^+^, ^96^Zr^+^, ^139^La^+^, ^140^Ce^+^, ^146^Nd^+^, ^147^Sm^+^, ^153^Eu^+^, ^155^Gd^+^, ^165^Ho^+^, ^159^Tb^16^O^+^, ^163^Dy^16^O^+^, ^166^Er^16^O^+^, ^169^Tm^16^O^+^, ^172^Yb^16^O^+^, ^175^Lu^16^O^+^, ^90^Zr_2_^16^O^+^, ^1806^Hf^16^O^+^, ^206^Pb^+^, ^207^Pb^+^, ^232^Th^16^O, and ^238^U^16^O^+^. Mount EKLP-5 did not include analyzes of ^7^Li^+^ though ^27^Al, or ^207^Pb. All analyses are performed using a single scan by peak-hopping cycles through the mass table, and each mass is measured on a single EPT® discrete-dynode electron multiplier operated in pulse counting mode. Count times for trace element measurements ranged from 1.5-12 sec/mass to optimize counting statistics for each isotope. The background for the electron multiplier it very low (<0.05 cps), and is statistically insignificant for the trace elements reported in this study.

The SHRIMP-RG was designed to provide higher mass resolution than other forward geometry large-format ion microprobe instruments (e.g., II Clement and Compston, 1994). The SHRIMP-RG geometry allows trace elements to be measured without the need to energy-filter the secondary beam and can be routinely measured without reducing the transmission of secondary ions to improve mass resolution. All measurements are made at mass resolutions of M/ΔM = ~10,000-10,500 (10% peak height), which eliminates interfering molecular species, particularly for ^45^Sc, ^48^Ti, and REE. Heavy rare earth elements (HREE) are measured as oxides because the metal ions contain isobaric interferences that cannot be fully resolved, which are not present for the oxide at higher mass. ^93^Nb requires mass resolution of ~14,325 (10% peak height) to fully resolve it from ^92^Zr^1^H, and is therefore, measured on the shoulder of the peak-flat to avoid the overlapping interference. At M/ΔM = 10,000 there is ~30% overlap between ^92^Nb and ^92^Zr^1^H, which likely represents a detection limit for low-Nb zircon of ~0.5 ppm. Higher Nb concentrations are robust.

Trace element concentrations for mounts EKLP-1 were standardized relative to a well-characterized, homogeneous zircon standards MAD-green (Barth and Wooden, 2010), which was mounted on a separate setup mount. Trace element concentrations for mounts EKLP-3 and EKLP-5 were calculated relative to MADDER – a well-characterized, homogeneous in-house zircon standards that is calibrated relative to MAD-green – which was co-mounted with unknowns on each mount. Ti concentrations were calculated relative to SL13 using Ti = 6.14 ± 0.01 (Hiess et al., 2008) for comparison with other published Ti in zircon values. The normalizing species for all trace element measurements was ^30^Si^+^. Interelement ratios were calculated uses the Microsoft Excel add-in programs Squid2.51 of Ken Ludwig (2009).

**References**

Barth AP, Wooden JL (2010) Coupled elemental and isotopic analyses of polygenetic zircons from granitic rocks by ion microprobe, with implications for melt evolution and the sources of granitic magmas. Chemical Geology 277: 149–159. doi:10.1016/j.chemgeo.2010.07.017.

Clement SWJ, Compston W (1994) Ion probe parameters for very high resolution without loss of sensitivity. Abstracts of the eighth international conference on Geochronology, cosmochronology, and isotope geology. U.S. Geological Survey Circular. U.S. Geological Survey. p. 384.

Hiess J, Nutman AP, Bennett VC, Holden P (2008) Ti-in-zircon thermometry applied to contrasting Archean metamorphic and igneous systems. Chemical Geology 247: 323–338. doi:10.1016/j.chemgeo.2007.10.012.

Hiess, J., Nutman, A. P., Bennett, V. C., and Holden, P., 2008, Ti-in-zircon thermometry applied to contrasting Archean metamorphic and igneous systems. Chemical Geology, 247, p. 323-338.

Ludwig, K.R., 2009, Squid 2, A user’s manual, Berkeley Geochronology Center Special Publication No. 5, p. 110.
